# Natural products targeting regulated cell deaths for adriamycin-induced cardiotoxicity

**DOI:** 10.1038/s41420-025-02389-w

**Published:** 2025-03-21

**Authors:** Zheng Wang, Yanli Zhu, Yu Yao, Wenyu Zhang, Bo Wang, Jing Wang, Yang Yang, Liwen Liu

**Affiliations:** 1 Xijing Hypertrophic Cardiomyopathy Center, Department of Ultrasound, Xijing Hospital, The Fourth Military Medical University, 127 Changle West Road, Xi’an, 710032 China; 2 Department of Cardiothoracic Surgery, Central Theater Command General Hospital of Chinese People’s Liberation Army, 627 Wuluo Road, Wuhan, 430070 China; 3 Xi’an Key Laboratory of Innovative Drug Research for Heart Failure, Northwest University First Hospital, Faculty of Life Sciences and Medicine, Northwest University, 229 Taibai North Road, Xi’an, 710069 China

**Keywords:** Heart failure, Drug development

## Abstract

Adriamycin (ADR), as an anti-cancer drug in routine clinical application, is utilized to treat various cancers such as ovarian cancer, hematological malignant tumor, and endometrial carcinoma. However, its serious dose-dependent cardiotoxicity extremely limits its clinical application. Currently, there remains a dearth of therapeutic agents to mitigate ADR-induced cardiotoxicity. Extensive research has demonstrated that ADR can simultaneously trigger various regulated cell death (RCD) pathways, such as apoptosis, autophagy, ferroptosis, necroptosis, and pyroptosis. Therefore, drugs targeting these RCD pathways may represent effective strategies for treating ADR-induced cardiotoxicity. Natural products, with their wide availability, low cost, and diverse pharmacological activities, have increasingly gained attention. Various natural products, including polyphenols, flavonoids, terpenoids, and alkaloids, can target the RCD pathways involved in ADR-induced cardiotoxicity. Furthermore, these natural products have exhibited excellent properties in preclinical studies or in vitro experiments. This review summarizes the mechanisms of RCD in ADR-induced cardiotoxicity and systematically reviews the natural products targeting these RCD pathways. Finally, we propose future research directions of natural products in this field.

## Facts

- The anti-cancer drug adriamycin (ADR) exhibits severe cardiotoxicity, significantly limiting its clinical application. Furthermore, there is a lack of therapeutic agents specifically targeting ADR-induced cardiotoxicity.
- Cell death is classified into accidental death and regulated cell death (RCD). The latter encompasses apoptosis, autophagy, ferroptosis, pyroptosis, and necrosis.
- ADR elicits cardiotoxicity by triggering multiple RCD pathways.
- A variety of natural products, including polyphenols, flavonoids, terpenoids, and alkaloids, exert protective effects by targeting the RCD pathways associated with ADR-induced cardiotoxicity.

## Open questions

- How does RCD pathway contribute to ADR-induced cardiotoxicity?
- Do natural products targeting RCD pathway exhibit protective efficacy against ADR-induced cardiotoxicity?
- How do natural products regulate the RCD pathway, such as autophagy, during different stages of disease progression?

## Introduction

Adriamycin (ADR) is one of the most typical and effective chemotherapeutic agents that is utilized for a wide range of cancers, such as ovarian cancer [1], hematological malignant tumor [2], and endometrial carcinoma [3]. Nevertheless, ADR has serious dose-dependent cardiotoxicity, which is irreversible and can gradually develop into arrhythmia or even heart failure [4]. These adverse reactions greatly restrict its clinical application. Dexrazoxane is the only drug currently approved for repressing ADR-induced cardiotoxicity. However, its long-term clinical application generates side effects [5]. Therefore, it is indispensable to investigate alternative pharmacotherapies for ADR-induced cardiotoxicity. Notably, natural products possess the advantages of a wide range of sources, inexpensive prices, and diverse pharmacological activities, attracting more and more attention. Importantly, several promising evidence from our group as well as some other laboratories illuminated that natural products also display immense potential in ameliorating ADR-induced cardiotoxicity [6, 7].

**Fig. 1:**
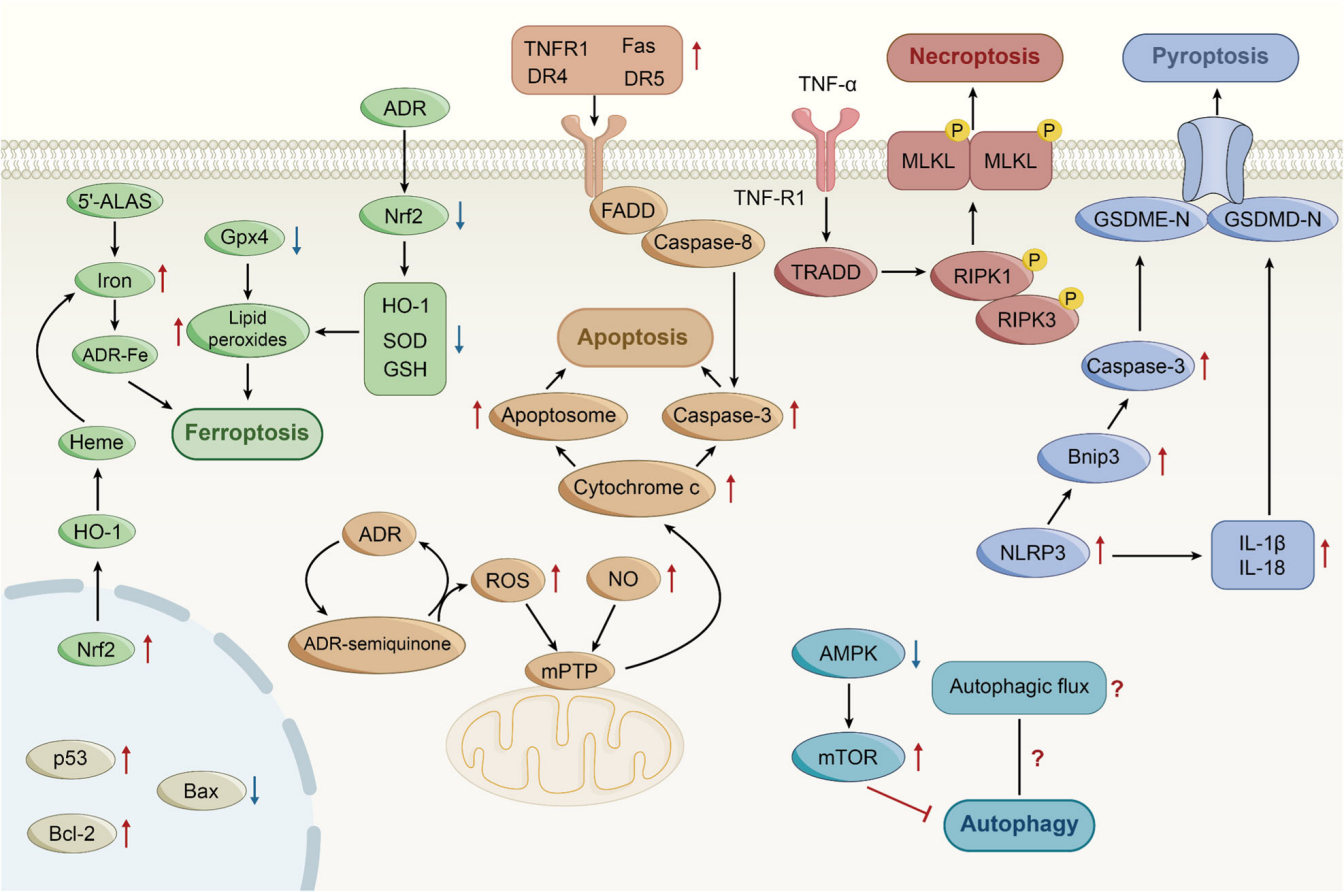
Schematic of the mechanisms of regulated cell deaths in ADR-induced cardiotoxicity. ADR, Adriamycin; 5′-ALAS, 5′-aminolevulinate synthase 1; AMPK, Adenosine monophosphate-activated protein kinase; Bcl-2, B-cell lymphoma-2; Bax, Bcl-2-associated X; DR, death receptor; FADD, Fas-associated with death domain protein; GSH, glutathione; Gpx4, Glutathione peroxidase 4; HO-1, heme oxygenase 1; IL, interleukin; SOD, superoxide dismutase; mPTP, mitochondrial permeability transition pore; mTOR, mammalian target of rapamycin; MLKL, mixed-lineage kinase domain-like protein; Nrf2, nuclear factor erythroid-2-related factor 2; NLRP3, NOD-like receptor family pyrin domain containing 3; NO, nitric oxide; RIPK1, receptor-interacting protein kinase 1; RIPK3, receptor-interacting protein kinase 3; TNF, tumor necrosis factor; TRADD, TNFR1-associated death domain; TNFR, TNF-receptor.

In the past decade, the Nomenclature Committee on Cell Death classified cell death into accidental death (ACD) and regulated cell death (RCD) [8]. RCD, which is controlled by delicate molecular signaling cascades and molecularly defined effector mechanisms, is crucial in the response to injury, infection, and inflammation [9]. Accumulating evidence has confirmed that ADR simultaneously triggers different RCD pathways, including previously established cell deaths (e.g., apoptosis, autophagy) [10, 11] and recently discovered (e.g., ferroptosis, pyroptosis) [12, 13] (Fig. 1). Remarkably, natural products perform protective functions against ADR-induced cardiotoxicity by modulating RCD pathway and may pave an emerging avenue for the prevention and treatment of ADR-induced cardiotoxicity (Table 1).

**Table 1:** A summary of protective effects of natural products on ADR-induced cardiotoxicity.

Natural products	Chemical Structure	Targets	Effects	Reference
Resveratrol		SIRT1 ↑; p53 ↓; AMPK ↑; Bcl-2 ↑; Bax ↓; S6K1 ↓; mTOR ↓; p62-Nrf2/HO-1 ↑	Inhibits apoptosis, regulates autophagy, and blocks ferroptosis	[28, 53–59]
Curcumin		caspase-3 ↓; Akt ↑; mTOR ↓; NLRP3 ↓; caspase-1 ↓ ; IL-18 ↓	Inhibits apoptosis, pyroptosis, and autophagy	[62–65]
Salvianolic acid A		Bcl-2 ↑; Bax ↓;	Blocks apoptosis	[68]
Luteolin		Bax ↓, caspase-3 ↓; caspase-9 ↓; Bcl-2 ↑	Inhibits apoptosis	[74–76]
Naringin		p38 ↓; MAPK ↓	Inhibits apoptosis	[81]
Pinocembrin		Nrf2 ↑; SIRT3 ↑ ; NLRP3 ↓	Inhibits pyroptosis	[82]
Dihydromyricetin		AMPK ↑; mTOR ↓	Induces autophagy	[83]
Spinacetin		SIRT3 ↑ ; AMPK ↑; mTOR ↓	Initiates protective autophagy	[84]
Fisetin		SIRT1 ↑; Nrf2 ↑; HO-1 ↑	Inhibits ferroptosis	[87]
Calycosin		ATG7 ↑ ; NLRP3 ↓ ; GSDMD ↑ ; IL-1β ↓ ; IL-18 ↓	Promotes autophagy and alleviates pyroptosis	[88, 90]
Daidzein		SIRT3 ↑; FOXO3a ↑	Represses apoptosis	[91]
Puerarin		PKCε ↑	Activates adaptive autophagy	[92]
Asiatic acid		Akt ↑; Nrf2 ↑	Suppresses apoptosis	[94]
Ursolic acid		Akt ↑; eNOS ↑	Prevents apoptosis	[95]
Corosolic acid		AMPKα2 ↑; mTORC 1 ↓	Promotes autophagy	[96]
Glycyrrhizin		HMGB1 ↓; Akt ↓; mTOR ↓	Improves autophagy	[98]
Rosmarinic acid		Fas L ↓	Represses apoptosis	[107]
Lycorine		Bcl-2 ↑; Bax ↓	Inhibits apoptosis;	[7]
Matrine		AMPKα/UCP2 ↑	Inhibits apoptosis;	[112]
Neferine		IGF-1R ↑; Nrf2 ↑	Inhibits autophagy	[113]
Berberine		Sirt3 ↑; SIRT1/p66 ↑	Inhibits apoptosis	[115, 116]
Rutaecarpine		Akt ↑; Nrf-2 ↑	Inhibits apoptosis	[118]
Psoralidin		Bcl-2 ↑; Bax ↓	Inhibits apoptosis	[6]
Epigallocatechin-3-gallate		AMPKα2 ↑ ; ULK1 ↑; mTOR ↓	Inhibits apoptosis and ferroptosis and activates adaptative autophagy	[120–122]
Cryptotanshinone		Akt ↑; GSK-3β ↑	Inhibits apoptosis	[124]
β‐LAPachone		mTOR ↓ and caspase‐3 ↓	Inhibits autophagy and apoptosis	[127]

Compelling evidence has indicated that many natural products play protective roles in ADR-induced cardiotoxicity by targeting regulated cell deaths, including apoptosis, autophagy, ferroptosis, necroptosis, and pyroptosis.

Some aspects of ADR-induced cardiotoxicity, including molecular mechanisms and mitochondrial-targeted therapy, have been well summarized previously [14, 15]. However, the emerging potential of natural products targeting RCD in ADR-induced cardiotoxicity has not been specifically summarized, and the present review aims to fill this gap. In this review, we first present the elaborate network of roles that RCD plays in ADR-induced cardiotoxicity. Subsequently, we highlight research progress on natural products in ADR-induced cardiotoxicity. Finally, some future directions and perspectives are suggested. This review presents the tremendous potential of natural products targeting RCD in ADR-induced cardiotoxicity and may provide more effective therapeutic strategies.

## The mechanisms of RCD in ADR-induced cardiotoxicity

### Apoptosis

Apoptosis represents one of the most classical cell death pathways and is also the most extensively explored in ADR-induced cardiotoxicity. ADR leads to excessive reactive oxygen species (ROS) production, transcription factors change, mitochondrial damage, peroxynitrite formation, which further stimulate the apoptotic signal, including both intrinsic and extrinsic apoptotic pathways [15–17].

The activation of the intrinsic pathway induced by ADR depends on the opening of the mitochondrial permeability transition pore (mPTP). After entering the cardiomyocytes, the anthraquinone structure of ADR will be catalyzed into a semiquinone metabolite with one electron and electrons are transferred to oxygen molecules, which finally leads to excessive ROS production [17]. Furthermore, the level of inducible nitric oxide synthase (iNOS) and nitric oxide (NO) in cardiomyocytes increases during ADR treatment [18]. All these events as well as the formation of the TOP II-ADR-DNA complex promote the opening of mPTP [19, 20]. Then, permeabilized mitochondria release cytochrome c to the cytosol [21], which leads to the formation of the apoptosome and the activation of caspase 3, eventually resulting in apoptosis [22]. In addition, ADR activates heat shock factor-1 and enhances the accumulation of heat shock protein-25 in the heart, further transactivating p53 that controls the transcription of many survivals as well as pro-apoptotic proteins, increasing the transcription of pro-apoptotic protein B-cell lymphoma-2 (Bcl-2)-associated X (Bax) [23].

ADR-induced apoptosis via the extrinsic pathway is driven by the death receptors. Zhao et al. illustrated that ADR treatment significantly increases all four death receptors (DRs), including tumor necrosis factor (TNF)-receptor (TNFR) 1, Fas, DR4, and DR5, at both mRNA and protein levels in the cardiomyocytes [24]. TNFR-associated death domain and Fas-associated death domain recruit caspase 8, and activated caspase 8 further activates caspase 3, ultimately accelerating apoptosis [22].

### Autophagy

Autophagy, a highly conserved catabolic process induced by cellular stress, interfaces with the regulation of core metabolism damage control and cell death, which can be either protective or detrimental depending on the specific cellular context [25, 26]. Multiple experiments have discovered disordered autophagy in response to the ADR challenge. However, it remains controversial whether ADR promotes or inhibits autophagy [27, 28].

Adenosine monophosphate-activated protein kinase (AMPK) and mammalian target of rapamycin (mTOR) signaling pathways play critical regulatory roles in Unc-51-like kinase 1 (ULK)-1 activity, thereby promoting and inhibiting autophagy, respectively [29]. ADR reduces basal phosphorylation of AMPK and its downstream target acetyl-CoA carboxylase, which activates mTOR signaling [30]. ADR also blocks autophagic flux in cardiomyocytes by impairing lysosome acidification and lysosomal function [27]. In contrast, Kobayashi et al. found that ADR markedly increases autophagic flux in cardiomyocytes, as reflected by the difference in protein levels of LC3-II (microtubule-associated protein light chain 3 form 2) or numbers of autophagic vacuoles [31]. The detailed causes of the above contradictory phenomenon may be dependent on the difference regarding the phase of autophagy, ADR dosage and duration, and other factors. Therefore, it is significant for researchers to intensively clarify the changes and mechanisms of cell deaths in different stages during ADR treatment, as it is closely related to the time of medical intervention to inhibit or activate related processes.

### Ferroptosis

Ferroptosis has provoked considerable attention since it was first described as an iron-dependent form of non-apoptotic cell death in 2012. It is driven by iron-dependent phospholipid peroxidation and is modulated by the extrinsic or transporter-dependent pathway and the intrinsic or enzyme-regulated pathway [32, 33]. Recently, it has been demonstrated to be implicated in ADR-induced cardiotoxicity.

Mitochondria-dependent ferroptosis is a central cause of ADR-induced cardiotoxicity. Glutathione peroxidase 4 (Gpx4), a selenocysteine-containing and glutathione-dependent enzyme, is a predominant regulator in ferroptosis. It catalyzes the reduction of specific lipid hydroperoxides into lipid alcohols, thereby reducing lipid peroxidation and further preventing ferroptosis [34]. Tadokoro et al. found that the ADR treatment remarkably downregulates both the mRNA and protein levels of total and mitochondrial Gpx4 and upregulates lipid peroxides in mitochondria. Gpx4 overexpression by adenovirus harboring mitochondrial Gpx4 increases Gpx4 in the mitochondria and effectively blunts ADR-induced ferroptosis [12]. Another RNA sequencing analysis also showed that ADR obviously curbs Gpx4 mRNA and protein levels [35]. In addition, ADR accumulated in the mitochondria in an mtDNA content-dependent manner by intercalating into mtDNA. ADR also decreased the abundance of 5’-aminolevulinate synthase 1(5’-ALAS), the rate-limiting enzyme in heme synthesis, thereby impairing iron utilization. Then, ADR-Fe complex formation in mitochondria triggered ferroptosis and subsequent cardiotoxicity [36].

In addition to the established roles of nuclear factor erythroid-2-related factor 2 (Nrf2) in maintaining proper redox homeostasis, it is not surprising that Nrf2 plays a critical role in ferroptosis as some downstream target genes of Nrf2, such as Gpx4 and heme oxygenase 1(HO-1) are ferroptosis-inducing agents [37]. Intriguingly, the roles of Nrf2 and its downstream molecules in ADR-induced cardiotoxicity are not uniform, which may be attributed to its multifaceted functions in different metabolism pathways. Fang et al. reported that ADR increased nuclear Nrf2 protein levels and enabled Nrf2 to promote HO-1 expression, thereby catalyzing heme degradation and facilitating the release of free iron, finally leading to ferroptosis and ultimately heart failure [38]. Conversely, in C57 mice and SD rat models of cardiotoxicity, the expression levels of antioxidant genes, such as HO-1, superoxide dismutase (SOD), and glutathione (GSH), are markedly decreased due to ADR-mediated inhibition of Nrf2 [39]. The findings by Li et al. also displayed similar results, indicating the cardio-protective role of targeting ferroptosis for cardiotoxicity prevention [35].

The aforementioned studies reveal that ADR leads to an increase in intracellular iron levels by reducing iron utilization in mitochondria or facilitating heme degradation and etc., ultimately inducing ferroptosis in cardiomyocytes.

### Necroptosis

ADR also triggers a form of programmed necrosis called necroptosis [40, 41]. Necroptosis is initiated by various cytokines and pattern recognition receptors, and cells dying by necroptosis manifest swelling and membrane rupture and release damage-associated molecular patterns, cytokines, and chemokines, thereby mediating inflammatory responses [41, 42]. Mechanistically, the TNF receptor recruits an early complex composed of TNFR1-associated death domain (TRADD) protein and receptor-interacting protein kinase 1 (RIPK1). RIPK1 and RIPK3 can form necrosome complexes that activate mixed-lineage kinase domain-like protein (MLKL) by a phosphorylation cascade, and phosphorylated MLKL undergoes oligomerization and migrates to the plasma membrane where it induces necroptosis by initiating membrane rupture or regulating ion [42, 43].

In ADR-treated mice, RIP3-induced activation of calcium calmodulin-dependent protein kinase II via phosphorylation or oxidation or both triggers the opening of mPTP and myocardial necroptosis. RIP3 deficiency protects the heart from ADR-induced alterations in cardiac contractility and morphology, as indicated by ameliorated myocardial necrotic death, fibrosis, and contractile dysfunction, identifying RIP3 as a promising target in ADR-induced cardiotoxicity [44]. Furthermore, the levels of phosphorylated RIP1 at serine 166/total RIP1 ratio, the phosphorylated RIP3 at serine 232/total RIP3 ratio, and phosphorylated MLKL at serine 358/total MLKL ratio are significantly augmented in ADR-treated rats, whereas donepezil (an acetylcholinesterase inhibitor) counteracts cardiotoxicity through reducing RIP1-mediated necroptosis [40]. A novel small molecular NADPH oxidase 2 (Nox2) inhibitor GSK2795039 has also been demonstrated to prevent RIP1-RIP3-MLKL-mediated cardiomyocyte necroptosis, improving myocardial remodeling and function in ADR-induced heart failure [45]. These suggest that the key molecules involving necroptosis may be the potential therapeutic target in ADR-induced cardiotoxicity.

### Pyroptosis

Pyroptosis is a form of lytic programmed cell death mediated by gasdermin. The activation of inflammasomes induced by various influencing factors induces the maturation of caspase-1 or caspase-4/5/11, both of which cleave gasdermin to release its N-terminal domain, which can bind membrane lipids and perforate the cell membrane [46, 47]. The gasdermin superfamily consists of gasdermin A/B/C/D/E (GSDMA/B/C/D/E) and DFNB59 (Pejvakin, PJVK) in human (Gsdma1-3, Gsdmc1-4, Gsdmd, Dfna5, and Dfnb59 in mice) [47]. Among these proteins, GSDMD and GSDME are the most deeply studied and also participate in ADR-induced cardiotoxicity.

ADR increased the levels of GSDMD-N and pro-interleukin (IL)-1β in a time-dependent manner and led to primary cardiomyocytes pyroptosis, as reflected by the rupture of the plasma membrane and the production of bubble-like vesicles [48]. Interestingly, the researchers further found that ADR not only induced the activation of inflammatory caspases and then indirectly regulated the production of GSDMD-N but also bound directly to GSDMD and promoted GSDMD-N-mediated pyroptosis, supporting GSDMD as a potential target for ADR-induced cardiotoxicity [48]. Notably, dilated cardiomyopathy patients exhibit hyper-activated NOD-like receptor family pyrin domain containing 3 (NLRP3) inflammasome with pyroptotic cell death of cardiomyocytes, which are negatively correlated with cardiac function [49]. Once NLRP3 inflammasome is activated, the mature forms of IL-1β and IL-18 release through the GSDMD pores, which may initiate and exacerbate inflammation and promote pyroptosis, finally leading to cardiac injury [49].

In addition, ADR also induced activation of caspase-3 and eventually triggered GSDME-dependent pyroptosis. Further investigation showed that ADR increased the expression of Bnip3, whereas silencing of Bnip3 blunted cardiomyocyte pyroptosis induced by ADR through regulating caspase-3 activation and GSDME cleavage, indicating that Bnip3-mediated caspase-3/GSDME signal was involved in ADR-induced cardiotoxicity [13].

In summary, ADR upregulates inflammatory cytokines, such as the expression of IL-1β, and induces the activation of apoptosis-associated protein caspases through various signaling pathways, promoting pyroptosis and eliciting cardiotoxicity.

## Natural products targeting regulated cell deaths in ADR-induced cardiotoxicity

### Polyphenol

Polyphenols are naturally occurring compounds that can be found in plants such as vegetables, fruits, whole grains, and nuts [50]. As the most abundant antioxidants in the human diet, they play beneficial roles in the prevention and the progress of chronic diseases such as diabetes and cardiovascular diseases, as well as aging [51]. In recent years, polyphenols have also been illustrated to exert a protective role in ADR-induced cardiotoxicity.

Resveratrol, a well-known polyphenol present in grapes and red wine, harbors various health benefits, including anti-oxidative, anti-inflammatory, and pro-angiogenic effects [52]. It has also been known to fight against ADR-induced cardiotoxicity through modulating RCD, such as apoptosis, autophagy, and ferroptosis. In 2011, Zhang et al. reported that the ADR-induced apoptotic index decreases from 11.8 to 7.0% after resveratrol treatment [53]. Resveratrol (incorporated into mouse standard chow at 0.01% (w/w)) not only upregulated silent information regulator 1 (SIRT1) and then resulted in the deacetylation of p53, finally reducing p53-mediated cardiomyocyte apoptosis but also inhibited the transcription-independent pro-apoptotic pathway of p53 through the release of cytochrome c [53]. Consistently, resveratrol (20 mg/kg/day) diminished ADR-induced cardiotoxicity in aged hearts by restoring SIRT1 activity, leading to the inhibition of USP7, a p53-deubiquitinating enzyme, followed by a decrement of p53 and Bax, which subsequently deactivated the apoptotic pathway [54]. AMPK, a key energy master, is another upstream regulator of p53 [55]. Resveratrol (25 µM) suppressed ADR-induced cardiomyocyte apoptosis via promoting AMPK phosphorylation and inhibiting P53 expression, as well as inducing Bcl-2 and downregulating Bax expression [56]. In addition to its inhibitory roles in apoptosis, resveratrol plays important regulatory effects in autophagy. After ADR treatment, the phosphorylation of S6K1, which can contribute to autophagy, is markedly elevated. Nevertheless, resveratrol (10 µM) prevented this result and inhibited autophagy, thereby attenuating ADR-induced cardiotoxicity [28]. Conversely, two other investigations confirmed that resveratrol improved ADR-induced cardiotoxicity by promoting autophagy [57, 58]. Specifically, resveratrol (20 µM) activated AMPK in a dose- and time-dependent manner, resulting in the phosphorylation/activation of ULK1 and the suppression of mTOR, which directly induced autophagy and possibly degraded harmful components and damaged cellular organelles [57]. The same group further found that resveratrol (10 mg/kg) significantly blocked the induction of E2F1/mTORC1 and E2F1/AMPKα2 pathway by ADR, leading to acceleratory autophagy and inhibitory apoptosis, thereby abolishing ADR-induced cardiotoxicity [58]. These paradoxical phenomena might be attributed to differences between these investigations in terms of cell lines, animal models, dosing and other components, which may call for intensive study in the future. Recently, Yu et al. explored the novel role and molecular mechanism of resveratrol in ADR-induced cardiotoxicity. Mechanistically, by upregulating the expressions of p62-Nrf2/HO-1, resveratrol remarkably blocked mitochondrial ROS overproduction and ferroptosis [59]. Notably, co-administration of resveratrol (diet containing 0.4% resveratrol) with ADR in young mice also restored the ability of the heart to undergo adaptive remodeling in response to hypertension later in life [60]. All the present studies shed light on the possibility that resveratrol may represent a possible therapeutic drug for ADR-induced cardiotoxicity by modulating cell deaths. More extensive research and exploration of experimental research and clinical development for resveratrol in cardiotoxicity are warranted in the future.

Curcumin, a crucial polyphenol present in *Curcuma longa* L. rhizome, has been applied as a pharmacological traditional medicinal agent in Ayurvedic medicine for about 6000 years [61]. Several studies have reported that curcumin can be suggested as a promising agent against ADR-induced cardiotoxicity by regulating cell deaths [62–65]. ADR-induced apoptosis is somewhat partly diminished by curcumin, which decreases the mRNA level of phosphate carriers and the activity of caspase-3 [62, 63]. 14-3-3γ is a member of the 14-3-3 protein family, which is implicated in coordinating multiple cellular processes, such as cell division, signal transduction, and apoptosis [66]. Notably, curcumin (50 mg/kg) obviously upregulated the expression of 14-3-3γ, which interacted with Bad in the cytoplasm, resulting in the translocation of Bcl-2 into the mitochondria. This blocked mPTP opening, and suppressed the formation of ROS-induced ROS release and excessive oxidative stress, which ultimately improved mitochondrial function and blunted apoptosis [64]. In addition, curcumin (100 and 200 mg/kg, mixed with 0.5% sodium carboxymethyl cellulose) inhibited pyroptosis and autophagy. Mechanistically, it increased phosphorylation of protein kinase B (Akt) and subsequent mTOR and partially reversed ADR-evoked changes in NLRP3, caspase-1, and IL-18 [65].

Besides, some other polyphenols exert protective effects in ADR-induced cardiotoxicity. Salvianolic acid A and Salvianolic acid B are both abundant bioactive compounds extracted from the Chinese traditional medicine *Salvia miltiorrhiza* [67]. Salvianolic acid A partially blocked apoptosis induced by ADR, as evidenced by a decreased ratio of TUNEL-positive cells, downregulated expression of pro-apoptotic proteins, and upregulated expression of anti-apoptotic protein Bcl-2. Mechanistically, Salvianolic acid A (10 µM and 50 µM) could inhibit the nuclear factor kappa-B (NF-κB) signaling induced by ADR, then downregulating LncRNA PVT1 expression [68]. Similarly, salvianolic acid B (20 µg/ml) pretreatment significantly attenuated ADR-induced myocardial apoptosis and ER stress by inhibiting TRPC3 and TRPC6-mediated intracellular Ca^2+^ overload [69]. In addition, tannic acid, punicalagin, and sinapic acid also suppressed apoptosis, thereby ameliorating ADR-induced cardiotoxicity [70–72]. In studies investigating the protective effects of tannic acid against ADR-induced cardiotoxicity, researchers divided male Sprague-Dawley rats into a control group, a ADR-treated group, a ADR combined with tannic acid-treated group, and a ADR combined with captopril-treated group. The results revealed that pretreatment with tannic acid (at doses of 20 and 40 mg/kg) significantly inhibited the ADR-induced elevation of pro-inflammatory cytokines TNF-α and IL-1β levels, while also reducing Bcl-2-like protein and caspase-3 activities, as well as c-fos and c-jun levels. These findings provide evidence that tannic acid attenuates ADR-induced cardiotoxicity by inhibiting inflammation and apoptosis [70]. Additionally, in studies exploring the protective mechanisms of sinapic acid against ADR-induced cardiotoxicity, researchers found that sinapic acid inhibited the expression of caspase-3 and Bax, and decreased the activities and levels of bcl-2-like protein and caspase-3, suppressing ADR-induced cardiotoxicity [72]. In summary, polyphenolic natural products can exert protective effects against ADR-induced cardiotoxicity by promoting or inhibiting autophagy and inhibiting cardiomyocyte apoptosis.

### Flavonoids

Flavonoids, abundantly present in commonly consumed fruits and vegetables, are highly bioactive compounds with very low toxicity and exert tremendous positive effects on health, which makes them attractive starting points in drug discovery [73]. Numerous types of research have indicated that flavonoids can be applied to prevent or treat ADR-induced cardiotoxicity by modulating cell deaths.

Flavone is a characteristic flavonoid widely distributed in natural plants with various bioactivities. Luteolin, as a natural flavone existing in vegetables, fruits, and herbs, markedly decreases the level of Bax, cleaved caspase-3, and caspase-9 and increases the levels of Bcl-2, serving protective effects in ADR-induced cardiotoxicity [74–76]. In line with this, baicalein (25 and 50 mg/kg for 15 days) and chrysin (25 and 50 mg/kg orally), which belong to trihydroxyflavone and hydroxyflavone, respectively, attenuated ADR-induced cardiotoxicity via repressing myocardial oxidative stress and apoptosis in mice [77, 78]. Furthermore, apigenin (25 mg/kg/day for 12 days via gavage) led to a significant decrease in caspase-3 and Bax and a remarkable enhancement in Bcl-2, thereby playing an anti-apoptotic effect [79]. Recently, Wei et al. found that wogonin derived from *Scutellaria baicalensis* protected rat hearts from ADR damage by serving an anti‑apoptotic role. TUNEL staining revealed that compared with the ADR-treated group, wogonin treatment (100 mg/kg via gavage) significantly ameliorated ADR-induced cardiomyocyte apoptosis in rats. The underlying mechanism involves inhibiting the release of cytochrome c from mitochondria and preventing the activation of caspase dependent apoptosis pathway [80]. Besides, naringin (1 µM) has also been reported to protect myocardial cells from ADR‑induced apoptosis, which may be achieved partially by inhibiting the p38/mitogen-activated protein kinases (MAPK) pathway [81]. The flavanone pinocembrin, which was originally isolated from honeybee propolis and rhizomes of the culinary herb Boesenbergia pandurate, dramatically improved cardiac contractile function, reduced cardiac fibrosis, and alleviated cardiac damage. The results from the vitro study indicated that pinocembrin (5 mg/kg every other day until the end of the study) markedly activated Nrf2/SIRT3 pathway to inhibit subsequent NLRP3-mediated pyroptosis [82].

Furthermore, some flavonols possessing antioxidant and anti-inflammatory capacities can improve ADR-induced cardiotoxicity. Dihydromyricetin is a flavanol extracted from *Hovenia dulcis* or *Ampelopsis grossedentata plants*. ADR dramatically inhibited the expression of p-AMPK and autophagy-related protein beclin-1 and LC3-II and also promoted activation of mTOR, whereas dihydromyricetin pretreatment (50 mg/kg or 100 mg/kg everyday) reversed these results, implying that dihydromyricetin induced autophagy to suppress ADR-induced cardiotoxicity by activating AMPK/mTOR axis [83]. Similarly, spinacetin (50 mg/kg or 100 mg/kg), an essential component in *Spinach*, alleviated ADR-induced cardiotoxicity by initiating protective autophagy through SIRT3/AMPK/mTOR pathways [84]. Nevertheless, rutin (100 mg/kg body weight) counteracted ADR-induced heart failure by inhibiting excessive autophagy [85]. In addition, a previous investigation found that fisetin (20 and 40 mg/kg) ameliorated ADR-induced cardiotoxicity via inhibition of oxidative stress, inflammation, and apoptosis [86]. Recently, it has been demonstrated to markedly alleviate cardiac dysfunction, myocardial fibrosis, and cardiac hypertrophy in rats. Mechanistically, fisetin increased the levels of SIRT1, Nrf2, and HO-1 and reversed the downregulation of Gpx4 expression and GSH level and the upregulation of ROS and malondialdehyde levels, whereas SIRT1 inhibition abolished the effects of fisetin. These indicated that fisetin (20 mg/kg/day and 40 mg/kg/day) attenuated ADR-induced cardiomyopathy by inhibiting ferroptosis through SIRT1/Nrf2 signaling pathway activation [87].

Isoflavone is another usual type of flavonoid. In zebrafish models, calycosin extracted from *Astragalus* attenuates ADR-induced cardiotoxicity via regulating apoptosis, autophagy, and pyroptosis [88–90]. Calycosin (1 µM, 10 µM, 50 µM) accelerated the accumulation of autophagosomes and autolysosomes and the knockdown of ATG7 decreased the number of autophagy-related structures, implying that calycosin promoted the formation of autophagosomes via ATG7 [88]. Recently, Zhang et al. confirmed that calycosin alleviated ADR-induced cardiotoxicity and pyroptosis by inhibiting NLRP3 inflammasome activation. Specifically, ADR dramatically resulted in the activation of NLRP3, ASC, and cleavage of caspase-1, GSDMD, IL-1β, and IL-18 in mice, whereas calycosin (5–160 µg/mL) counteracted the above results [90]. Daidzein, an isoflavone found in soy foods, effectively inhibited ADR-induced heart failure by repressing inflammation, fibrosis, apoptosis, and oxidative stress and regulating energy metabolism through the SIRT3/FOXO3a pathway [91]. Puerarin (80 µM), an isoflavone extracted from the root of *Pueraria lobata*, increased 14-3-3γ expression, which phosphorylated protein kinase C ε (PKCε) and impelled PKCε to translocate on mitochondria, thus activating adaptive autophagy [92].

The aforementioned studies indicate that flavonoid natural products can exert protective effects against ADR-induced cardiotoxicity by anti-apoptosis, inhibiting pyroptosis, suppressing ferroptosis, inducing autophagy, and inhibiting excessive autophagy.

### Terpenoids

Terpenes are the largest class of small-molecule natural products on earth and are widely distributed in various plants [93]. Previous studies have indicated that almost all terpenes, including triterpenes, sesquiterpene, monoterpenes, and diterpenes, can be used to treat ADR-induced cardiotoxicity by inhibiting apoptosis.

Asiatic acid is an urthane pentacyclic triterpene in *Centella Asiatica*. It is reported that asiatic acid (10 mg/kg or 30 mg/kg) protects against ADR-induced cardiomyopathy via activating Akt signaling, which restores Nrf2 activation and suppresses oxidative damage and apoptosis to improve cardiac function [94]. Similarly, after ADR treatment, ursolic acid (100 µL DMSO (80 mg/kg/day)), which presented in many medicinal plants, preserved cardiac function and decreased cardiac cell apoptosis. Mechanistically, ursolic acid upregulated the phosphorylation levels of Akt and endothelial nitric oxide synthase (eNOS) and repressed eNOS uncoupling induced by ADR, which led to enhanced NO levels and decreased ROS production, preventing cardiac cell apoptosis [95]. In addition to apoptosis, some other natural products restore autophagic flux to prevent ADR-induced cardiotoxicity. For example, the urthane pentacyclic triterpene corosolic acid (10 mg/kg or 20 mg/kg) isolated from *Lagerstroemia speciosa* L increased the expression of p-ULK1 and Beclin1, promoting autophagosomes formation and autophagolysosomes degradation, which is achieved by activating AMPKα2-mTORC 1 signaling pathway [96]. Yoon et al. demonstrated that the lupinane pentacyclic triterpenes betulinic acid (0.1–1 µM) derived from betulin decreased protein expression levels of Bax and cleaved caspase-3/-9 while increasing the expression of Bcl-2 [97]. Glycyrrhizin is an oleanane bioactive triterpenoid saponin extracted from the traditional Chinese medicinal herb *Glycyrrhiza uralensis Fisch*. Glycyrrhizin pretreatment (25 mg/kg/d or 50 mg/kg/d) improved autophagy flux via high mobility group box 1 (HMGB1)-dependent Akt/mTOR pathway to prevent ADR-induced cardiotoxicity [98].

Some sesquiterpenes, monoterpenes, and diterpenoids, in addition to triterpene, are also reported to mitigate ADR-induced cardiotoxicity via regulating cell deaths. β-Caryophyllene, a natural bicyclic sesquiterpene abundantly present in essential oils from various spices, has been approved by the United States Food and Drug Administration and European agencies as a food additive [99]. β-Caryophyllene treatment (25 mg/kg) in ADR-injected rats exhibited a downregulated expression of pro-apoptotic proteins (Bax, p53, and caspase-3) and upregulated expression of the anti-apoptotic protein Bcl-2 in the myocardium, implying that β-caryophyllene played its potent anti-apoptotic property in ADR-injected rats [100, 101]. Likewise, sesquiterpene, including curdione and nerolidol, as well as monoterpene such as geraniol and citronellal, attenuated oxidative stress and apoptosis in ADR-induced cardiotoxicity [102–105]. Rosmarinic acid, mostly derived from Rosmarinus officinalis, Salvia officinalis, and Perilla frutescens, is outlined as potent anti-bacterial, antioxidative, anti-proliferative, and anti-nociceptive agent [106]. Zhang et al. showed that ADR enhanced the release of soluble Fas L from cardiac fibroblast, which in turn facilitated cardiomyocyte apoptosis. However, rosmarinic acid treatment (100 mg/kg/d) counteracted the induction and release of Fas L. Rosmarinic acid treatment also inhibited the activation of nuclear factor of activated T and matrix metalloproteinases-7 expression, which is crucial in rosmarinic acid-mediated suppression of Fas L and protective effect on cardiomyocyte apoptosis [107]. Additionally, the diterpenoid Kirenol and Ginkgolide B exerted cardioprotective properties against ADR-induced cardiotoxicity by impeding apoptosis [108, 109]. Diterpenoid kirenol (15 µmol/mL) enhanced the activation of Bcl-2 and Bcl-xL, inhibiting caspase-dependent apoptosis [108]. Gao et al. pretreated rat H9c2 cardiomyocyte cells with ginkgolide B followed by ADR treatment. Their findings revealed that pretreatment with ginkgolide B (1 µM, 5 µM, and 50 µM) significantly reduced apoptosis in H9c2 cells by decreasing ROS and intracellular calcium levels and activating Akt phosphorylation [109].

In summary, terpenoid natural products can exert protective effects against ADR-induced cardiotoxicity by inhibiting apoptosis-related signaling pathways, reducing the expression of pro-apoptotic proteins, and improving autophagy flux.

### Alkaloids

Alkaloids derived from plants, serving as a rich source for drug discovery, are important natural agents for promoting healthcare and disease prevention [110]. Our team has focused on lycorine, which is a natural piperidine alkaloid extracted from *Amaryllidaceae* and possesses a range of pharmacological activities, including the regulation of autophagy and the induction of cancer cell apoptosis [111]. Notably, we also demonstrated that lycorine (2.5 mg/kg, 5 mg/kg or 10 mg/kg) decreases ADR-induced cardiotoxicity via inhibiting cardiomyocyte apoptosis, as evidenced by remarkably increased Bcl-2/Bax levels [7]. Matrine, a natural piperidine extracted from the root of Sophora flavescens, ameliorated ADR-induced H9c2 cell apoptosis and oxidative stress level. Researchers exposed mice to ADR to establish a mouse model of ADR-induced cardiotoxicity, with a saline-treated control group established for comparison. Subsequently, H9c2 cells from these mice were used to verify the in vitro effects of matrine. The results showed that ADR triggered an increase in reactive oxygen species (ROS) production and excessive apoptosis in cardiomyocytes. Matrine (200 mg/kg/day) alleviated ADR-induced apoptosis and elevated oxidative stress levels in H9c2 cells by activating AMPKα/UCP2, suggesting its potential as a promising therapeutic agent for ADR-induced cardiotoxicity [112]. Priya et al. observed that overwhelmed ROS with a concomitant decrease in cellular antioxidant status of H9c2 cardiomyoblasts by ADR exposure promotes autophagy by suppression of IGF-1R signaling and Nrf2 pathway. However, neferine (10 µM) isolated from the seed of Nelumbo nucifera Gaertn was able to activate the Nrf2 pathway and enhance the expressions of HO-1 and SOD to modulate ADR-mediated regulation of IGF-1R survival signaling [113].

Additionally, Berberine is a representative isoquinoline alkaloid as well as an eminent component of traditional Chinese medicine for more than 2000 years [114]. It also confers protection against ADR-induced cardiotoxicity, which partially depends on apoptosis [115, 116]. In studies investigating the mechanisms of berberine’s protective effects against ADR-induced cardiotoxicity, researchers found that overexpression of Sirtuin 3 (Sirt3), a mitochondrial deacetylase that regulates the activity of proteins involved in apoptosis, autophagy, and metabolism, helped reduce DOX cytotoxicity in H9c2 cardiomyocytes. Berberine (1 µM and 10 µM) acted as a modulator of Sirtuin function and cellular quality control pathways to mitigate DOX toxicity [115]. In another study, berberine (10 and 20 mg/kg) protected the heart from DOX damage through SIRT1-mediated inhibition of the 66 kDa Src homology 2 domain-containing protein (p66Shc) [116]. Similarly, the isoquinoline lotusine (10 µM and 50 µM) from *Nelumbo nucifera* mitigated ADR-mediated apoptosis by downregulating the levels of Bax and caspase-3 [117]. Rutaecarpine, a quinazolinocarboline alkaloid that is extracted from the traditional Chinese *herb Evodia rutaecarpa*, inhibited ADR-induced enhancement of malondialdehyde as well as apoptosis. The researchers further illustrated that rutaecarpine (20 mg/kg or 40 mg/kg) activated Akt or Nrf-2, which further upregulated the antioxidant enzymes such as HO-1 and GSH cysteine ligase modulatory subunit expression, thereby fighting against ADR-induced cardiotoxicity [118].

In conclusion, alkaloids may be drug candidates in the progression of ADR-induced cardiotoxicity. Future research focusing on more rigorous clinical studies of the most promising alkaloids and the further exploitation of recently discovered candidate alkaloids may facilitate their clinical development.

### Others

In addition to the compounds previously mentioned, there are many other natural agents with potential protective effects in ADR-induced cardiotoxicity via regulating cell deaths. For example, our team has shown the protective effects and mechanisms of psoralidin, a natural phenolic coumarin isolated from the seeds of the medicinal plant *Psoralea corylifolia* L. ADR downregulated the expression of Bcl-2 and upregulated the expression of Bax. However, psoralidin pretreatment (25 mg/kg) reversed these effects [6]. Green tea is the most widely consumed beverage besides water and has attracted considerable attention due to its health benefits [119]. Epigallocatechin-3-gallate (EGCG), one of the major bioactive esters in green tea, play cardioprotective roles in ADR-induced cardiotoxicity by alleviating apoptosis [120, 121]. Researchers evaluated the protective effects of EGCG against ADR-induced cardiotoxicity. Their findings revealed that ADR-induced apoptosis was manifested by increased levels of NF-κB, tumor suppressor protein p53, calpain 2, and caspases 3 and 12. Pretreatment with EGCG (40 mg/kg) significantly reduced these apoptotic signals, exerting a protective effect against ADR-induced cardiotoxicity [120]. In another study exploring the protective mechanisms of catechins against ADR-induced cardiotoxicity in adult male albino rats, researchers pretreated the rats with 400 mg/kg of catechins for 2 weeks before administering 1.66 mg/kg of ADR. The results showed that ADR treatment upregulated the expression of heart injury markers such as lactate dehydrogenase (LDH), creatine kinase (CK), and creatine kinase-MB (CK-MB), and these changes were reversed in the catechin-treated group [121]. Noticeably, EGCG pretreatment (20 µM) can also dramatically diminish iron accumulation, counteracted oxidative stress and abnormal lipid metabolism, and thereby alleviated ADR cardiotoxicity-induced ferroptosis [122]. Furthermore, EGCG pretreatment (20 µM) considerably activated the AMPKα2-ULK1 axis and inhibited the AMPKα2-mTOR axis, thereby activating adaptative autophagy and protecting cardiomyocytes against ADR-induced cardiotoxicity [122].

*Salvia miltiorrhiza*, a well-known traditional Chinese medicine, also named Danshen in China, is used not only in human medicine but also in health-promotion food. Cryptotanshinone and Tanshinone IIA, two major components of *Salvia miltiorrhiza*, protect against cardiotoxicity induced by ADR in vitro and in vivo [123, 124]. Cryptotanshinone (10 µM and 25 µM) increased cell viability, reduced ROS levels, inhibited apoptosis, and protected mitochondrial membrane integrity via the Akt-glycogen synthase kinase 3β (GSK-3β)-mPTP pathway [124]. Chrysophanol is an anthraquinone compound isolated from the rhizome of *Rheum palmatum* L [125]. Lu and colleagues illustrated that chrysophanol (5 mg/kg/day, 20 mg/kg/day, and 40 mg/kg/day) suppressed the effects that ADR significantly increases cardiac apoptosis, mitochondrial injury, and cellular PARylation levels [126]. β‐LAPachone (5 mg/kg), a natural quinone obtained from *Handroanthus impetiginosus*, reduced levels of p‐mTOR and caspase‐3 in a dose‐dependent manner and elevated the levels of beclin‐1, leading to improved autophagy and apoptosis [127]. Interestingly, quinones exhibited anti-proliferation and anti-metastasis effects in various cancer types [128]. Therefore, the future exploration of natural products which both possess anti-proliferation effects and cardio-protective roles will be significant as this can achieve reducing toxicity and enhancing efficacy.

From the evidence presented above, natural products such as polyphenols, flavonoids, terpenoids, alkaloids, and quinones exhibit considerable therapeutic potential in ADR-induced cardiotoxicity by regulating various apoptosis-related signaling pathways and the expression of related molecules.

## Future directions and perspectives

ADR is one of the most used anti-tumor chemotherapy drugs in the clinic. However, its cardiotoxicity severely restricts its application. At the same time, no specific therapy is developed so far for direct prevention and treatment of ADR induced-cardiotoxicity, partly because the current understanding of the exact mechanisms and hence relevant therapeutic targets is not sufficient. Fortunately, certain promising findings indicate the potential direction of future research and may be valuable for the treatment of cardiotoxicity. Next, we will focus on potential future research directions regarding natural products in ADR-induced cardiotoxicity.

Recent research on the treatment of ADR induced-cardiotoxicity in natural products is mainly concentrated on the monomers, and only a few are compound pharmaceuticals. Notably, Fatease et al. discovered that the use of combined polymer micelles in ovarian cancer quercetin/resveratrol and resveratrol/curcumin not only achieved chemical sensitization but also reduced heart toxicity both in mice when challenged with ADR doses known to cause acute toxicity and in a chronic ADR treatment model [129–131]. Importantly, the combination of multiple compounds is one of the useful approaches to overcome the limited application of natural products by enhancing their absorption and bioavailability. For instance, a novel berberine-glycyrrhizic acid complex formulation modulates the pharmacokinetics of berberine and increases berberine plasma concentration, thereby enhancing the prevention effect [132]. Due to the complex cardiotoxic process involving cell deaths, a single activation or inhibition of the related mechanisms is likely to fail to achieve clinical treatment. Therefore, the combination of two or more natural products may have a better effect on cardiotoxicity in ADR, which may also be related to its targeting multiple pathways. In the future, with increasing applications of molecular biological techniques for combinatorial chemistry approaches, there will be broad prospects for combined treatment of multiple natural ingredients and exploring drug delivery systems targeting multiple processes in ADR-induced cardiotoxicity.

Non-coding RNA received great attention in various fields, and related research has even subverted the traditional point of view. Non-coding RNA has been proven to be inextricably linked with the life processes such as organ fibrosis, aging, and metabolism [133–135]. Importantly, non-coding RNA can not only be used as a biomarker of ADR-induced cardiotoxicity [136, 137], but also interact with a variety of signal pathways mentioned above to act as a promoter or inhibitor in ADR-induced cardiotoxicity. However, there are few studies on the role of RNA regulated by natural products in ADR-induced cardiotoxicity. Guo et al. found that irigenin isolated from the rhizome of *belamcanda chinensis* showed significant efficacy in terms of ADR-induced cardiotoxicity by partly suppressing apoptosis via the increase of miR-425, as demonstrated by the upregulation in Bcl-2, and downregulation in Bax, cleaved Caspase-3 and PARP compared with ADR-treated mice [138]. Therefore, the future investigation of emerging associations between natural products and non-coding RNA may open up a new field of therapeutic and diagnostic opportunities for ADR-induced cardiotoxicity.

Although pharmacological treatments regarding natural products to prevent ADR-induced cardiotoxicity are well-established in animal models, research continues to be plagued by the inability to translate research findings into clinically useful therapies. Noticeably, a pilot clinical study showed that astragalus polysaccharide may be an effective therapy for preventing cardiotoxicity induced by epirubicin [139]. Furthermore, natural products described above, including resveratrol and curcumin, have been applied to clinical trials in other diseases [140–143]. In the future, it is expected that more reliable clinical evidence (e.g., randomized controlled trials) will be provided, which will also help to promote the clinical application of natural drugs against ADR-induced cardiotoxicity.

## Conclusions and discussion

ADR-induced cardiotoxicity is common and inevitable in the process of anti-tumor. Many natural products have shown excellent protection against ADR-induced-cardiotoxicity in preclinical studies or due to their extensive pharmacological mechanisms. Meanwhile, researchers are looking for future drugs, whether in traditional medicinal plants or in plant-derived natural compounds, which are important resources for complementary and alternative medicine.

Numerous studies have reported that autophagy exerts opposite effects during the early and late stages of pathogenesis, indicating the presence of a time-dependent mechanism. For example, Mattiolo et al. found that sustained autophagy during short-term starvation enhanced apoptosis. Conversely, during long-term starvation, autophagy exhibited a pro-survival effect [144]. Similarly, a study exploring the role of autophagy during the repair phase following proximal tubular ischemia/reperfusion (I/R) injury found that inhibiting autophagy exacerbated renal dysfunction in the early stage of acute kidney injury (AKI), whereas it alleviated renal injury during the AKI repair phase [145]. The use of the autophagy inhibitor 3-MA exacerbated renal tissue apoptosis and I/R injury, while the activation of autophagy with the natural compound rapamycin mitigated renal I/R injury [146]. Likewise, in hypoxia/reoxygenation (H/R), autophagy is an early response to hypoxia and is further induced in the early phase of reoxygenation. However, as the duration of reoxygenation increases, autophagy gradually decreases, while apoptosis peaks. The activation of autophagy with the natural product rapamycin can reduce cellular apoptosis [146]. These findings suggest that autophagy occurs in a time-dependent manner during different stages of disease progression. Targeted drug therapy at different stages of disease progression may improve treatment success rates.

Palmitic acid (PA) exerts dual effects on autophagy by inducing autophagy and blocking autophagic flux. Zheng et al. treated human hepatocellular carcinoma (HepG2) cells with PA and found that PA increased the expression of microtubule-associated protein light chain 3-II (LC3-II)/actin, indicating the activation of autophagy. However, prolonged PA stimulation reduced the expression of autophagy-related markers, suggesting that autophagic flux was restricted [147]. Similarly, propolis also has dual effects on autophagy. Studies have found that the combination of ADR and geopropolis inhibits autophagy in human monocytes by suppressing cytosolic LC3 levels, alleviating ADR-induced adverse reactions in cancer treatment [148]. Interestingly, in an investigation of the anti-proliferative and anti-inflammatory effects of propolis in the human melanoma cell line A375, researchers examined autophagy markers in A375 cells. The results showed that propolis treatment induced autophagy in A375 cells by decreasing the LC3-I/LC3-II ratio while increasing the expression of the autophagy-related gene (Atg) 5/Atg12 complex and p62 protein [149]. Furthermore, phenolic compounds are largely known for their antioxidant properties by inhibiting the production of ROS and autophagy. However, at high concentrations, phenolic compounds, especially flavonoids, disrupt intracellular redox balance through mitochondrial membrane depolarization, thereby promoting ROS production and inducing cellular oxidative stress and autophagy [150].

Numerous studies have confirmed that different concentrations of ROS can promote or inhibit cellular autophagy. Physiological levels of ROS can inactivate phosphatidylinositol 3,4,5-trisphosphate 3-phosphatase to activate PI3K, and subsequently activate autophagy, promoting cell survival [151]. In contrast, high levels of ROS continuously engage in irreversible reactions with lipids, proteins, and DNA, leading to cellular oxidative damage and death. Additionally, excessive ROS has also been shown to inhibit autophagy and promote cell apoptosis [152]. Therefore, the dual effects of natural products on autophagy may depend on their impact on intracellular ROS levels.

In conclusion, studies focusing on the role of natural products in cardiotoxicity have significantly enriched our understanding of the prevention and treatment of cardiotoxicity caused by ADR. Before the natural products are successfully applied to clinical treatment for inhibiting ADR-induced cardiotoxicity, extensive work is urgently required.
